# Impulsive and Omission Errors: Potential Temporal Processing Endophenotypes in ADHD †

**DOI:** 10.3390/brainsci11091218

**Published:** 2021-09-15

**Authors:** Johan E. Acosta-López, Isabel Suárez, David A. Pineda, Martha L. Cervantes-Henríquez, Martha L. Martínez-Banfi, Semiramis G. Lozano-Gutiérrez, Mostapha Ahmad, Wilmar Pineda-Alhucema, Luz M. Noguera-Machacón, Moisés De La Hoz, Elsy Mejía-Segura, Giomar Jiménez-Figueroa, Manuel Sánchez-Rojas, Claudio A. Mastronardi, Mauricio Arcos-Burgos, Jorge I. Vélez, Pedro J. Puentes-Rozo

**Affiliations:** 1 Facultad de Ciencias Jurídicas y Sociales, Universidad Simón Bolívar, Barranquilla 080005, Colombia; cervantesmh@unisimonbolivar.edu.co (M.L.C.-H.); mmartinez108@unisimonbolivar.edu.co (M.L.M.-B.); lozanosemiramis@gmail.com (S.G.L.-G.); mostapha.ahmad@unisimonbolivar.edu.co (M.A.); wpineda1@unisimonbolivar.edu.co (W.P.-A.); lnoguera1@unisimonbolivar.edu.co (L.M.N.-M.); mdelahoz48@unisimonbolivar.edu.co (M.D.L.H.); emejia18@unisimonbolivar.edu.co (E.M.-S.); gdjimenez@unisimonbolivar.edu.co (G.J.-F.); sanchezr@unisimonbolivar.edu.co (M.S.-R.); ppuentes1@unisimonbolivar.edu.co (P.J.P.-R.); 2 Universidad del Norte, Barranquilla 081007, Colombia; delchiaroi@uninorte.edu.co; 3 Neuropsychology and Conduct Research Group, University of San Buenaventura, Medellín 050010, Colombia; david.pineda1@udea.edu.co; 4 INPAC Research Group, Fundación Universitaria Sanitas, Bogotá 111321, Colombia; mastronardic@hotmail.com; 5 Grupo de Investigación en Psiquiatría (GIPSI), Departamento de Psiquiatría, Instituto de Investigaciones Médicas, Facultad de Medicina, Universidad de Antioquia, Medellín 050010, Colombia; 6 Grupo de Neurociencias del Caribe, Universidad del Atlántico, Barranquilla 081007, Colombia

**Keywords:** ADHD, endophenotypes, temporal processing, reaction time, genetics, Caribbean community, heritability, precision medicine

## Abstract

Temporal processing (TP) is associated with functions such as perception, verbal skills, temporal perspective, and future planning, and is intercorrelated with working memory, attention, and inhibitory control, which are highly impaired in individuals with attention deficit hyperactivity disorder (ADHD). Here we evaluate TP measures as potential endophenotypes in Caribbean families ascertained from probands affected by ADHD. A total of 232 individuals were recruited and clinically evaluated using an extensive battery of neuropsychological tasks and reaction time (RT)-based task paradigms. Further, the heritability (genetic variance underpinning phenotype) was estimated as a measure of the genetics apportionment. A predictive framework for ADHD diagnosis was derived using these tasks. We found that individuals with ADHD differed from controls in neuropsychological tasks assessing mental control, visual-verbal memory, verbal fluency, verbal, and semantic fluency. In addition, TP measures such as RT, errors, and variability were also affected in individuals with ADHD. Moreover, we determined that only omission and commission errors had significant heritability. In conclusion, we have disentangled omission and commission errors as possible TP endophenotypes in ADHD, which can be suitable to assess the neurobiological and genetic basis of ADHD. A predictive model using these endophenotypes led to remarkable sensitivity, specificity, precision and classification rate for ADHD diagnosis, and may be a useful tool for patients’ diagnosis, follow-up, and longitudinal assessment in the clinical setting.

## 1. Introduction

Attention deficit hyperactivity disorder (ADHD) is a complex neurodevelopmental disorder that affects up to 5–16% of children and adolescents worldwide [1,2,3,4,5,6,7]. The Diagnostic Statistical Manual of Mental Disorders [8] specifies the existence of three different Presentation of ADHD, namely predominantly inattentive, predominantly hyperactive-impulsive, and combined type. Several studies based on multigenerational families, adoption, and twin studies, have provided compelling evidence that genetic factors contribute to a substantial portion of the ADHD phenotypic variance [9]. In particular, first and second degree relatives of patients with ADHD have markedly higher prevalence of symptoms [10].

Given the highly variable clinical manifestations of ADHD, it has been suggested that endophenotypes might be a better approach to dissect genetic and neurophysiological basis of ADHD. The endophenotype concept refers to markers that are more directly connected to neuropsychological, behavioral, or neuroanatomical components associated with the disease [11,12,13]. Endophenotypes might be defined as “measurable components”: (1) associated with the disorder occurring at a higher frequency in affected individuals than in the general population; (2) heritable and state-independent (that is, manifest in individuals whether the illness is active); (3) co-segregating with the disease through generations within families; and (4) enriched in the causal pathway between genes and disease [13,14,15,16]. Endophenotypes overcome the inherent difficulties of a symptoms-based clinical diagnosis, and have the potential to differentiate between potential diagnoses that present with similar symptoms [17,18], which make them suitable to study the genetic and neurophysiological basis of ADHD. It is crucial that endophenotypes are easily measurable with standardized strategies for their quantification and evaluation [19].

Several ADHD studies have identified potential cognitive endophenotypes in neuropsychological tasks [11,20,21,22], including dysfunctions in response inhibition, sustained attention, working memory, and motor control [23]. However, the cognitive mechanism boiling down all those cognitive dysfunctions is the temporal processing (TP), which is one of the principal measures reported to be affected in patients diagnosed with ADHD when different tasks and paradigms are used [11,23,24,25].

TP is associated with functions such as perception, verbal skills, temporal perspective, and future planning. These time-dependent functions are intercorrelated with other impaired functions in ADHD such as working memory, attention, and to a lesser degree inhibitory control. These key timing deficits appear to survive when these functions are controlled, suggesting independent cognitive deficits in the temporal domain [26]. Strong evidence in time deficits and behavioral measures of impulsivity and inattention suggests that timing issues are key to the clinical behavioral profile of ADHD [27]. TP is classically measured using reaction time (RT) paradigms, which provide an interesting context to study different measures of TP. 

RTs are defined as the elapsed time (usually in milliseconds [ms]) between the presentation of a stimulus until the reaction or response and constitute a measure of information processing. Errors of commission provide an index of faster information activation (i.e., impulsivity or attention) [28], while omission errors and variability are classically related as indexes of sustained attention, respectively [28]. During RT-based paradigms, it is classically observed that faster TP increases the number of errors of commission while more omissions arrived at the longest RT [29].

In addition to the aforementioned deficits, deficiencies in the perception and subjective appreciation of time are equally relevant [26,27]. ADHD affected individuals show difficulties in their inner clock due to failures in sequential continuity awareness, reproduction and perception of time, leading to overly fast operation in standby periods. Thus, TP is an important neuropsychological component that has previously been proposed as an ADHD endophenotype [30,31,32]. In this regard, greater impairment of TP is found ADHD inattentive individuals [26]. Other authors found that individuals with ADHD take longer to complete tasks than healthy controls, showing a greater discrepancy between the completion times of the expected objectives [33]. Likewise, the use of TP tasks provides evidence of anticipatory and/or impulsive responses, due to failures in the discrimination of short RT intervals [34,35,36,37], which suggests that ADHD affected are prone to a greater number of commission errors. Overall, ADHD affected individuals exhibit TP dysfunction, at least as far as temporal reproduction is concerned [38].

To our knowledge, there are no reports of family-based studies in Caribbean communities, especially in those of significant African admixture, that investigate different measures of TP as possible cognitive endophenotype in patients diagnosed with ADHD. Here we explored the definition of TP endophenotypes in a sample of 232 individuals belonging to 67 nuclear families from Barranquilla, Colombia, the biggest city of the Northern Colombian Caribbean coast, who were clinically characterized using neuropsychology batteries as well as TP measures using RT-based tasks. Our overarching hypothesis was that there were specific patterns of TP and the existence of different endophenotypes in this community that represent a significant variance of the ADHD symptomatology, which could play an important role in neuropsychological models of TP [39,40,41,42] and help to better understand the etiology of this neuropsychiatric condition [10,39,40,41,42,43,44,45]. To shed some light into the role of these neuropsychological and TP measures on ADHD diagnosis, a predictive model of ADHD status that includes sex, age and potential RTs endophenotypes as predictors was constructed.

## 2. Subjects and Methods

### 2.1. Subjects

We recruited 232 individuals from 67 nuclear families segregating ADHD with at least a single ADHD-affected individual, from patients attending our research program in ADHD advertised in the Grupo de Neurociencias del Caribe’s website [46]. All individuals participated voluntarily and provided informed written consent either directly or from their parents. Initially, 124 nuclear families with at least one child affected with ADHD were sequentially ascertained. However, because of the nature of the RT task (see below), only full families with children aged 6 years and above were included. Most families were of medium socioeconomical strata and inhabit the city of Barranquilla and its metropolitan area in the Atlántico Department located at the Northern Colombian Caribbean coast. Barranquilla’s population is the result of a racial admixture between Aboriginal Amerindian communities with Spaniards and Africans, and later with Syrians-Lebanese, Sephardi Jews, Germans, Italians, and English immigrants [47,48]. The admixture composition of this community (~63% African with a vast Amerindian contribution) [49] suggests an ethnic heterogeneity [50,51] that substantially differs from that of the “Paisa” community, a genetic isolate from Colombia with a high prevalence of ADHD and a very small African ethnical background [52,53,54,55,56]. This study was approved by the Ethics Committee of Universidad Simón Bolívar at Barranquilla, Colombia (approval # 00032 of 13 October 2011).

### 2.2. Clinical Assessment

#### 2.2.1. ADHD Diagnosis

We performed an extensive clinical, neurological, and neuropsychological evaluation to define ADHD status and the presence of other comorbidities as described elsewhere [20,57,58,59]. Briefly, we employed the Diagnostic Interview for Children and Adolescents version IV (DICA-IV) [60,61,62] as the gold standard to assess the diagnosis of ADHD and/or ADHD comorbidities (i.e., conduct disorder [CD] and oppositional defiant disorder [ODD]) in children, adolescents and adults. For children and adolescents, the DICA-IV structured interview was completed by their parents who reported children’s symptoms and consequences in the academic, legal and work-related areas, as well as alcohol and tobacco consumption, and its consequences [60,62,63]. Persistent symptoms impacting family, social and work-related environments were also recorded. Following the C criteria of DSM-IV, ADHD symptoms in children and adolescents were evaluated by their parents and teachers using the Colombian version of the Behavioural Assessment System for Children (BASC) [64] and the ADHD checklist [65,66].

#### 2.2.2. Neuropsychological Assessment

##### Stroop’s Color and Word Test

This test assesses individual’s ability of selective attention (i.e., inhibitory control) and requires individuals to suppress automatic responses in favor of a specific response requested by the evaluator [67]. The test consists of three paper sheets: (1) administration sheet: a list of 100 words corresponding to the color’s names red, blue and green written in black ink; (2) color sheet: 100 sets of four letters “X”; and (3) interference sheet: indicates the color-word administration condition and contains 100 words with colors’ names (red, blue and green) written in color ink that mismatch the color of the actual word [68,69,70].

##### Cross-Out-Squares Test

Similar to Toulouse-Pierrón test [71], the cross-out-squares test (COST) assesses sustained attention with nonverbal stimulation. It consists in presenting 140 squares with a line in different positions in one of the angles or sides of each square. Individuals should cross-out as fast as possible squares that were equal to the stimuli at the top of the sheet. The total number of correct cross-out squares (maximum 48 points), as well as the number of omission and commission errors and the time to complete the task are quantified. In individuals with ADHD, these variables reflect difficulties at inhibitory level [72].

##### Trail Making Test (TMT)

This is a paper-and-sheet test with two parts. In Part A, numbers 1 to 25 are randomly presented in a paper sheet. Individuals should connect, as fast as possible, the numbers in consecutive order using a straight line (that is, 1-2-3-...-25). In Part B numbers 1 to 13 and letters A to L are randomly in a paper sheet. The task consists of connecting, alternating in consecutive order, numbers and letters (i.e., 1-A-2-B-3C-4D-5E-6F-…-13-L) [72,73,74,75,76,77].

#### 2.2.3. Reaction Time Tasks Assessment

##### Conner’s Continuous Performance Test-II (CPT-II)^®^

This is a computerized test in which stimuli consists of letters presented every 250 ms [78,79,80]. A total of 360 letters in six blocks are randomly presented. The time interval of stimuli presentation (1, 2 or 4 s) varies within blocks. Individuals are told to press the spacebar when all letters but letter “X” are shown. The total completion time of the CPT is 14 min and can only be administered to individuals aged 6 years or more [81].

##### Go/No-Go Tasks

We administered a two-part Go/No-Go (GNG) task using visual signals [82,83]. Part A consists of 50 randomized trials of 2000 ms long and 1000 ms inter stimulus interval, with a usual GNG naturalistic dominant stimuli (green [go] and red [no-go] signals). Individuals had to react the Go signal (green light) and had to stop the response to the known No-Go signal (red light). Part B consists of 50 trials of reversal Go/No-Go stimuli (red [go] and green [no-go] signals) with the same randomized and automatically scored conditions of Part A. Here, the Go signal was changed to red light and the No-Go or stop signal was changed to green light [83]. RTs were obtained and recorded for both Part A and Part B using a multi-operational apparatus for reaction times (MOART) system model 35600, with PsymSoft II Psychomotor Control Software model 35800. Individuals performed the GNG tasks between 8 and 11 am, in a light-, temperature-, and noise-controlled environment [82,83].

### 2.3. Statistical Analysis

Means and standard deviations (SDs) were used to summarize numerical variables. These variables were compared among ADHD affected and unaffected individuals using two-sided Mann–Whitney *U* test for two independent samples. Uncorrected Cohen’s *d* was calculated to measure the effect size for all variables [84,85]. To avoid the effect of potential confounding variables such as age and sex, *p*-values were corrected using analysis of covariance (ANCOVA). For categorical variables, frequencies and proportions were calculated; comparisons were performed using a χ^2^ test of independence. All statistical analyses were performed in R version 3.4.3 (https://www.R-project.org/, accessed on 6 December 2017).

### 2.4. Heritabilty Estimation

Heritability is defined as the proportion of the total phenotypic variation explained by genetic factors [86]. To estimate the heritability of neuropsychological and RT-based variables in our set of families, we used the ASSOC module in the Statistical Analysis of Genetic Epidemiology (SAGE) software [86,87,88], which evaluates the association between a continuous trait and one or more covariates from pedigree data in the presence of familial correlations, and simultaneously estimates familial variance components (i.e., familial correlations and heritability) [87]. Parameters in the segregation model evaluated by ASSOC are estimated by maximum likelihood under the assumption that all parameters, including polygenic heritability and other familiar correlations, follow multivariate normality [87,88,89,90].

### 2.5. Predictive Model for ADHD

Following previous work by our group and others [20,91,92,93,94,95], we applied Advanced Recursive Partitioning Approach (ARPA) using the Classification and Regression Tree (CART) [96,97] and TreeNet [98] modules implemented in the Salford Predictive Modeller^®^ software suite (Salford Systems, San Diego, CA, USA; https://www.salford-systems.com/, accessed 10 December 2017) to construct a predictive tree-based model of ADHD status in our cohort. A short description of CART, Random Forest, and TreeNet is provided elsewhere [20,91,92,94,95]. Sex, age, and potential TP endophenotypes were used as predictors. ARPA is widely used in predictive analyses as (1) it accounts for non-linear hidden interactions, (2) is independent of the type of data and of the data distribution type, (3) offers fast solutions to reveal hidden complex substructures and (4) provides non-biased statistical analyses of high dimensional data [99]. The performance of this predictive model was assessed by calculating the Receiver Operating Characteristic (ROC) curve and the area under the ROC curve (AUC), as well as different performance measures for binary classifiers including the sensitivity (*S_e_*), specificity (*S_p_*), and correct classification rate (accuracy), among others (Supplementary Material).

## 3. Results

### 3.1. Subjects

Two-hundred and thirty-two individuals (104 [44.8%] females, 128 [55.2%] males) from 67 nuclear families were included in this study (Table 1). From this group, 124 (53.4%) individuals were diagnosed with ADHD (40 [32.3%] females, 84 [67.7%] males). No children or adults were under medication for ADHD. The male-to-female ratio was 2.1 (95%CI = 1.46–3.16) among ADHD affected individuals; as expected, the ADHD diagnosis distribution differed by sex (χ^2^ = 15.94, degrees of freedom [*df*] = 1, *p* = 6.53 × 10^−5^). The mean ± SD for age at diagnosis in the whole sample was 28.24 ± 15.45 (range: 8–60); statistically significant differences were found by ADHD status (affected: 23.13 ± 15.6; not affected: 34.11 ± 13.06) but not by sex (female: 28.99 ± 13.88; male: 27.64 ± 16.64, *W* = 6715.5, *p* = 0.9075). The average family size was 3 ± 0.64 individuals (range 3–6); a total of 40 (59.7%) trios, 24 (35.8%) quartets, two (3%) families with five members, and one (1.5%) family with six members were recruited. Of note, this sample differs from that used in previous studies of ADHD in this Caribbean Community [20,59,70] because the CPT can only be applied to individuals aged 6 years or more.

### 3.2. Differences in TP Neuropsychological Tests between Affected and Unaffected Individuals

**Figure 1:**
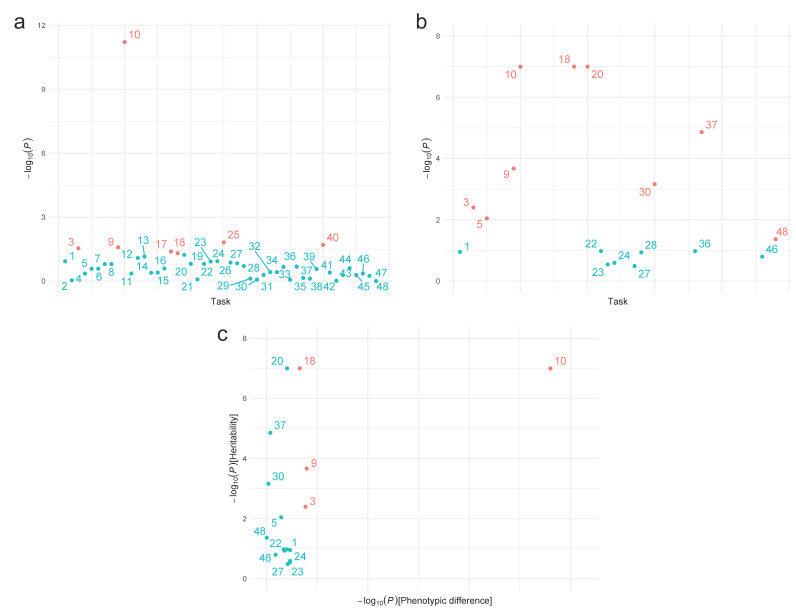
Neuropsychological tasks where (**a**) ADHD affected individuals differed from controls; (**b**) genetic effects and hereditary transmission are present; and (**c**) fulfil the requirements to be considered as potential endophenotypes are shown in orange. Displayed numbers correspond to task # in Table 2.

Our main results are presented in Table 2. After controlling for age and sex, we found statistically significant differences between ADHD affected and unaffected individuals in tasks of temporal processing of information, which assess the RT and individual’s performances. In particular, ADHD affected individuals showed more omission errors than unaffected individuals in MOART-based tasks (0.27± 0.64 vs. 0.07 ± 0.3, *p* = 0.029) and COST (6.32 ± 5.71 vs. 4.77 ± 4.34, *p* = 6.02 × 10^−12^), and more lecture errors in the Stroop test (1.3 ± 2.01 vs. 0.6 ± 1.12, *p* = 0.049) (Figure 1a), which refer to the number of incorrectly identified colors. We also found differences between ADHD affected and unaffected individuals in the typical scores of the omission and commission errors (Punctuation 1; 62.22 ± 32.63 vs. 58.48 ± 20.17, *p* = 0.015) and perseverance (Punctuation 8; 60.77 ± 23.63 vs. 58.6 ± 22.16, *p* = 0.020) of the CPT. Furthermore, individuals with ADHD obtained a lower hit rate than unaffected individuals in the COST (41.62 ± 5.71 vs.42.88 ± 4.48, *p* = 0.026) (Figure 1a).

### 3.3. Heritability Estimates

We found strong statistical evidence supporting hereditary transmission (i.e., heritability parameter, *h*^2^) in neuropsychological tests assessing RT in our sample (Table 2 and Figure 1b). These variables include the omissions in Part A (*h*^2^ = 0.349, *p* < 0.004) and Part B (*h*^2^ = 0.369, *p* < 0.009) of the GNG task, the number of hits (*h*^2^ = 0.626, *p* < 2.14 × 10^−4^) and omissions (*h*^2^ =0.66, *p* < 1 × 10^−7^) in the COST, and the lecture ( =0.705, *p* < 1 × 10^−7^) and denomination errors (*h*^2^ = 0.661, *p* < 1 × 10^−7^) in the Stroop test. We also found that the RT prepotent response standard deviation (PRSS) (*h*^2^ = 0.499, *p* < 1.38 × 10^−5^) and Punctuation 3 (*h*^2^ = 0.506, *p* < 6.87 × 10^−4^) of CPT’s Part B are heritable. Similarly, Punctuation 12 (*h*^2^ = 0.246, *p* < 0.043) of the HSEISICH showed evidence genetics effects and hereditary transmission. No genetics effects and hereditary transmission in the Trail Making Test were found (Table 2 and Figure 1b).

### 3.4. Potential Endophenotypes

Following our results, the omission errors in the GNG task Part A, the number of hits and omissions in the COST as well as the number of lecture errors of the Stroop test, can be considered TP endophenotypes in ADHD (Table 2 and Figure 1c).

**Table 2:** Performance of 232 individuals from the Colombian Caribbean on TP and RT-based neurological and neuropsychological tasks.

#	Task	Affected (*n* = 124)	Unaffected (*n* = 108)	*d*	*p*	Heritability	
						*h*^2^ (SD)	*p*
	**MOART**						
	*Part A* (Go)	**Mean (SD)**	**Mean (SD)**				
1	RT (ms)	532.54 (130.41)	472.22 (87.7)	0.536	0.118 *^b^*	0.188 (0.154)	0.111
2	Commission errors	0.95 (1.39)	0.52 (1.09)	0.340	0.929 *^b^*	*^a^*	*^a^*
*3*	Omission errors	0.27 (0.64)	0.07 (0.38)	0.362	**0.029** *^b^*	0.349 (0.131)	**0.004**
4	Early responses	1.46 (2.64)	0.59 (1.51)	0.400	0.445	*^a^*	*^a^*
	*Part B* (No-Go)						
5	RT (ms)	513 (119.63)	463.84 (97.32)	0.448	0.264 *^b^*	0.369 (0.157)	**0.009**
6	Commission errors	1.4 (1.93)	0.69 (1.29)	0.430	0.263 *^b^*	*^a^*	*^a^*
7	Omission errors	0.31 (0.77)	0.11 (0.37)	0.320	0.160 *^b^*	*^a^*	*^a^*
8	Early responses	2.48 (4.33)	0.83 (1.76)	0.485	0.159 *^b^*	*^a^*	*^a^*
	**Cross-out-squares test**						
9	Hits	41.62 (5.71)	42.88 (4.48)	0.244	**0.026**	0.626 (0.178)	**2.14 × 10^−4^**
*10*	Omissions	6.32 (5.71)	4.77 (4.34)	0.303	**6.02 × 10^−12^** *^b^*	0.66 (0.106)	**<1 × 10^−7^**
11	Commissions	1.76 (5.6)	1.82 (5.08)	0.011	0.440 *^b^*	*^a^*	*^a^*
12	RT (ms)	209.35 (112.12)	156.67 (64.32)	0.567	0.082 *^b^*	*^a^*	*^a^*
	**Trail Making Test**						
13	Hits in *Part A*	23.62 (0.81)	23.89 (0.31)	0.426	0.071	*^a^*	*^a^*
14	RT in *Part A* (ms)	54.02 (64.61)	38.33 (21.86)	0.317	0.410 *^b^*	*^a^*	*^a^*
15	Hits in *Part B*	22.43 (3.32)	22.96 (2.05)	0.189	0.398	*^a^*	*^a^*
16	RT in *Part B* (ms)	128.28 (91.06)	96.4 (64.05)	0.401	0.254 *^b^*	*^a^*	*^a^*
	**Stroop test**						
	*Lecture*						
17	RT (ms)	76.94 (48.01)	60.43 (20.42)	0.438	**0.041** *^b^*	*^a^*	*^a^*
*18*	Errors	1.3 (2.01)	0.6 (1.12)	0.419	**0.049** *^b^*	0.705 (0.088)	**<1 × 10^−7^**
	*Denomination*						
19	RT (ms)	124.64 (76.05)	96.85 (49.74)	0.427	0.059 *^b^*	*^a^*	*^a^*
20	Errors	3.77 (4.43)	2.4 (4.19)	0.318	0.155	0.661 (0.086)	**<1 × 10^−7^**
	*Mismatch*						
21	RT (ms)	112.4 (80.55)	95.8 (70.05)	0.219	0.827 *^b^*	*^a^*	*^a^*
22	Errors	6.32 (6.35)	3.75 (5.29)	0.437	0.155 *^b^*	0.207 (0.165)	0.105
	**CPT**						
23	Omissions	14.66 (15.88)	8.09 (9.89)	0.490	0.121 *^b^*	0.1 (0.178)	0.287
24	Omissions (%)	4.55 (4.92)	2.5 (3.07)	0.492	0.117 *^b^*	0.119 (0.179)	0.254
25	Punctuation 1	62.22 (32.63)	58.48 (20.17)	0.136	**0.015** *^b^*	*^a^*	*^a^*
26	Commission	20.55 (14.93)	13.24 (7.84)	0.602	0.134 *^c^*	*^a^*	*^a^*
27	Commission (%)	53.74 (22.68)	36.71 (21.58)	0.768	0.147 *^c^*	0.076 (0.165)	0.323
28	Punctuation 2	51.02 (8.36)	49.41 (8.01)	0.196	0.196	0.2 (0.167)	0.116
29	Average hit RT (ms)	479.32 (83.98)	477.7 (66.43)	0.021	0.764	*^a^*	*^a^*
30	Punctuation 3	61.34 (10.92)	62.77 (9.5)	0.139	0.851 *^b^*	0.506 (0.158)	**6.87 × 10^−4^**
31	Standard deviation of hit RT (ms)	10.84 (7.03)	8.23 (3.97)	0.449	0.513 *^b^*	*^a^*	*^a^*
32	Punctuation 4	57.17 (12.13)	58.37 (9.93)	0.107	0.376 *^b^*	*^a^*	*^a^*
33	Variability	20.71 (20.55)	12.95 (10.48)	0.467	0.380 *^b^*	*^a^*	*^a^*
34	Punctuation 5	56.48 (12.27)	56.75 (9.7)	0.024	0.216 *^b^*	*^a^*	*^a^*
35	Detectability	0.5 (0.68)	0.69 (0.4)	0.347	0.856 *^b^*	*^a^*	*^a^*
36	Punctuation 6	52.09 (8.37)	50.22 (8.75)	0.219	0.210	0.203 (0.162)	0.105
37	PR standard deviation	0.95 (0.94)	1.05 (1.17)	0.095	0.714 *^b^*	0.499 (0.119)	**1.38 × 10^−5^**
38	Punctuation 7	53.76 (12.73)	53.5 (11.69)	0.021	0.765	*^a^*	*^a^*
39	Perseverations	7.48 (14.21)	2.33 (4.66)	0.475	0.272 *^b^*	*^a^*	*^a^*
40	Punctuation 8	60.77 (23.63)	58.6 (22.16)	0.094	**0.020** *^b^*	*^a^*	*^a^*
41	HRTBCH	0.01 (0.03)	0 (0.02)	0.104	0.399 *^b^*	*^a^*	*^a^*
42	Punctuation 9	49.77 (9.85)	49.68 (9.94)	0.009	0.979	*^a^*	*^a^*
43	HSEBCH	0.06 (0.1)	0.04 (0.07)	0.251	0.527 *^b^*	*^a^*	*^a^*
44	Punctuation 10	50.56 (12.31)	53.14 (11.23)	0.218	0.248	*^a^*	*^a^*
45	HRTISICH	0.07 (0.07)	0.04 (0.05)	0.456	0.517 *^b^*	*^a^*	*^a^*
46	Punctuation 11	50.49 (12.76)	47.08 (11.93)	0.276	0.445 *^b^*	0.163 (0.163)	0.159
47	HSEISICH	0.09 (0.12)	0.06 (0.09)	0.300	0.566 *^b^*	*^a^*	*^a^*
48	Punctuation 12	49.87 (10.3)	48.4 (10.97)	0.139	0.992 *^c^*	0.246 (0.144)	**0.043**

^*a*^ Parameter could not be maximized in SAGE. ^*b*^ Corrected for age using ANCOVA. ^*c*^ Corrected for sex and age using ANCOVA. CPT: Conners’ continuous performance test; *d*: Cohen’s effect size; *h*^2^: heritability estimated value; HRTBCH: Hit RT to block change; HSEBCH: Hit RT standard error to block change; HRTISICH: Hit RT interstimulus interval; HSEISICH: Hit standard error of the interstimulus interval; MOART: Multi-operational apparatus for reaction times; PR: Prepotent response; RT: Reaction time; SD: Standard deviation. *p*-values < 0.05 are shown in **bold**. Task numbers in *italics* are included in the predictive model (see Figure 1).

### 3.5. Predictive Model for ADHD Diagnosis

**Figure 2:**
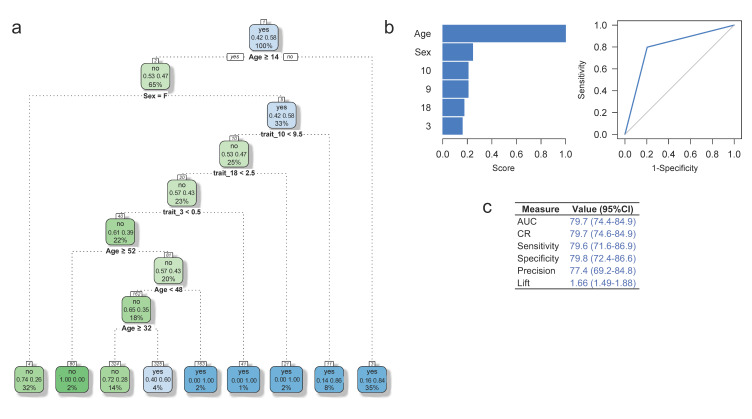
(**a**) Classification tree for predicting ADHD status in individuals from the Colombian Caribbean. Numbers within white squares represent the node number, the first line corresponds to the most frequent class (no: unaffected; yes: ADHD affected), the second line to the probability of each class within the node, and the third line to the percentage of the total sample size (*n* = 232) within each node. Nodes where ADHD affected individuals are more likely to be classified are shown in **blue**. (**b**) Variable importance (left) and ROC curve for the CART strategy. Displayed numbers correspond to task # in Table 2. (**c**) Performance measures for the learning (blue) data set; the grey line represents a naïve classifier. AUC: Area under the curve; CART: Classification and regression tree; CI: confidence interval; CR: Classification rate; ROC: Receiver operating characteristic.

A seven-level tree with seven terminal nodes was derived by CART to differentiate ADHD affected from unaffected individuals in our cohort (Figure 2a). Splitting nodes include age at diagnosis, sex and traits 3 (omissions in the GNG task), 10 (omissions in COST), and 18 (lecture errors in Stroop test). It is noteworthy that these variables defining splitting nodes were also found to be endophenotypes (see Table 2 and Figure 1c). This predictive model yields sensitivity, specificity and correct classification rate (yields *S_e_*, *S_p_* and accuracy values) values of 79.6% (95% CI = 71.6–86.9%), 79.8 % (95% CI = 72.4–86.6%) and 79.7% (95% CI = 74.6–84.9%), respectively, suggesting that these RT endophenotypes allow the accurate prediction of ADHD status in our cohort. Although this CART-based predictive model yields similar results when validated using RF and TreeNet (data not shown), it outperforms that including only sex and age to predict ADHD status (Supplementary Material).

Our sample includes 232 clinically evaluated individuals (node 1, 58% ADHD affected; Figure 2a). In the first split, children aged 14 or younger (35% of the total sample) have 84% chance of being ADHD affected regardless of sex (terminal node 3). Individuals in node 5 (33%, *n* = 232), that is, males aged 14 years or older, have a 58% chance of being diagnosed with ADHD. This chance increases to 86% when 9.5 or more omissions in the COST are made (18% of the total sample, node 11, *n* = 18). Twenty-five percent of the sample (*n* = 57) is in node 10 and have a 53% chance of being diagnosed as ADHD unaffected.

In node 4, 32% of the sample (*n* = 73) are females aged 14 years or older, and have 74% of being ADHD unaffected (Figure 2a). Node 20 comprises 23% of the total sample (*n* = 52); these individuals are male aged 14 years or less that made less than 2.5 lecture errors in the Stroop test and less than 9.5 omissions in the COST. Node 40 gathers 51 male individuals (22% of the total sample) aged more than 14 but less than 48 years that made <0.5 omissions in the GNG tasks, <2.5 lecture errors in the Stroop test and <9.5 omissions in the COST. Individuals with these characteristics have a 61% chance of being diagnosed as ADHD unaffected.

## 4. Discussion

In this study, we aimed to further investigate measures of temporal processing (TP) as possible cognitive endophenotype in ADHD. Different measures of TP were assessed in 232 individuals from nuclear families segregating ADHD from a Caribbean Community in Barranquilla, Colombia and dissect potential TP endophenotypes. TP was measured using RT-based paradigms such as the CPT and Go/No-Go tasks. Additionally, neuropsychological assessment was carried out using instruments such as the Stroop test, the cross-out-squares test (COST) and the Trail Making test (TMT) parts A and B (Table 2).

First of all, we identified important phenotypic differences between ADHD-affected and unaffected individuals in the TP measures (Table 2) [100]. In the CPT task, individuals with ADHD committed more omission and commission errors than controls; similar results were observed for the number of commission errors in the COST task. Omission errors in TP are one of the most reported findings in RT-based studies [67,101,102]. This type of error is associated with the absence of accelerated responses, has been proposed as one of the main components of the gradual and trial-by-trial variability [67,101,103,104,105] and is considered responsible for the fast (or slow) but ineffective performance fluctuations typical in individuals with ADHD [103,104,106,107]. Furthermore, omission and commission errors are associated with occasional lapses as well as fluctuations and/or neural oscillations in circuit calibration during timely repetitive tasks [108]. Evidence suggests that this non-response may be due to an underestimation of time intervals resulting in a delay in information processing [109]. In this sense, alterations are mainly related to activation, control in the preparation and/or pre-adjustment of the motor response [110]. Thus, deficits in motor response processing and preparation make it difficult to perform tasks involving sustained effort, activation and use of time, which lead to a diminished performance that is sufficiently severe to produce omission errors [108,111].

We found that ADHD affected individuals differed from unaffected individuals in omissions and perseverance of the CPT (Table 2). Omissions represent a deficit in stimulus detection, which leads to difficulties to sustain a correct response. Children with ADHD are more imprecise than unaffected children due to problems in vigilance and sustained attention, which lead to an increased number of incorrect responses [112]. Perseverance reflects a slow reaction to problems in the selection criterion, and an inappropriate engagement control. Altogether, perseverance yields difficulties in the mobility of cognitive resources when permanent changes occur due to compromised alertness, sustained and selective attention, as well as difficulties with time-limited testing in ADHD [113]. This suggests a series of slower and more variable responses to visually presented stimuli, which lead to failures in the simultaneous processing of a second stimulus in ADHD affected individuals [114]. On the other hand, the differences found in errors of commission (Table 2) are in line with those proposing this trait as an ADHD-specific TP deficit [110]. Another aspect is that slower information processing and the higher variability in the RT could produce more omission errors which highlights the existence of differences in execution performance between ADHD affected and unaffected individuals [31,115,116,117]. Most importantly, these measures are heritable, thus commission and omission errors can be considered TP endophenotypes in ADHD (Table 2 and Figure 1).

It is noteworthy that the COST showed a differential pattern between ADHD affected and unaffected individuals in our sample (Table 2). Indeed, ADHD affected individuals have a lower number of hits compared to unaffected individuals. This difference is associated with precision difficulties in visual-search tasks and slow processing rates, which lead to a lower speed of processing information [118]. The speed of visual processing is related with omission errors and has been found to be different between ADHD affected and unaffected individuals [119,120].

Our findings also indicate that ADHD affected individuals committed more lecture errors in the Stroop task compared to unaffected individuals and this measure was also heritable and there is a tendency for ADHD affected to spend more time to complete the task (Table 2). This diminution in the speed across phases may be due to the occurrence of conflicts in information processing (i.e., errors) [121], leading to difficulties in the changes from the most automatic to the least automatic tendencies [122]. Some studies indicate that this phenomenon is related to a low sensitivity and difficulties for hit-error-hit changes [123,124,125,126], manifested in an alternation of the excitability and inhibition, as well as in the motor sensorial control. These alterations might be associated with cortical hypoactivation [127,128,129,130] and functional connectivity reduction [131].

In addition to genetic aspects, the aforementioned neuropsychological alterations have been proposed as working models in ADHD for the development of more precise diagnostic tools, and to obtain better treatment responses [11,31,132,133]. Erroneous responses and RT variability are important for the identification of intermediate phenotypes useful in genetic molecular studies of ADHD [134,135,136]. Finding strong evidence of heritability in several neuropsychological variables assessing RT and errors (Table 2) is consistent with previous studies, indicates an important genetic component in RT and neuropsychological features in families segregating ADHD [133,137,138,139], and supports future genetic association studies of TP endophenotypes in our cohort.

The idea of a TP deficit in ADHD is coherent with different models, where TP is a complex cognitive process that involves multiple components to modulate time-associated tasks. In ADHD, TP highlights the importance of time perception and task-specific demands and coincides with current ADHD models. The ARPA-based predictive model of ADHD diagnosis includes, in addition to demographic variables such as age and sex, three TP endophenotypes derived from neuropsychological instruments used to assess TP (Table 2 and Figure 2). This predictive model performs reasonably well in terms of sensitivity, specificity, correct classification rate, the area under the ROC curve and lift, which makes it a plausible alternative for the diagnosis of ADHD compared to the DSM-IV criteria. Considering that (1) impulsive and omission errors are associated with occasional lapses in attention (i.e., failures of sustained attention that are severe enough to produce an error of omission) [140,141,142,143,144], (2) using RT-based task helps to establish differential variability patterns between ADHD affected and unaffected individuals [103,104,140,141,142,143,144] associated to fluctuations in fast and/or slow, but ineffective responses (i.e., errors of omission and/or commission) [106,107], this predictive model could be used in the clinical practice for ADHD diagnosis and follow-up, as well as to contribute to personalized interventions by elucidating the clinical heterogeneity of the disorder in this Caribbean community.

Future research directions could potentially include the study of TP measures, RT variability and the trial-by-trial performance variability using entropy-based measures and transitions between states (i.e., correct-incorrect-correct responses and early response followed by timeouts) that might be useful to better understand, from a different perspective, the differential evolution of pattern of individuals with ADHD when compared to controls. Using our family-based cohort, these findings could be subsequently integrated to complex segregation, and genetic linkage and association studies of TP towards the development of more precise and differential ADHD diagnosis [31,133,137,145,146]. Linkage and association genetic studies could, in parallel, help to establish TP and RT-based endophenotypes as neurobiological markers in ADHD that could contribute to better understand its etiology [21] and support the contribution of the *ADGRL3*, *SNAP-25*, *FGF1* and *DRD4* genes, which were previously reported to be associated with ADHD [4,5,147,148,149,150,151,152]. In the future, these results might be important to improve the attention, characterization, diagnosis and follow-up of individuals with ADHD through translational and precision medicine approaches [112,153,154,155].

## Figures and Tables

**Table 1:** Demographic characteristics of 232 individuals included in this study.

	Unaffected	Affected	Statistic Index	*p*	Cohen’s Effect Size
	(*n* = 108)	(*n* = 124)			
Sex	Frequency (%)	Frequency (%)	*χ* ^2^		
Female	64 (59.25)	40 (32.26)	15.942	<0.00001	-
Male	44 (40.75)	84 (67.74)			
	Mean (SD)	Mean (SD)	Mann–Whitney’s *U*		
Age	34.11 (13.06)	23.14 (15.60)	9239.5	<0.0001	0.758

## Supplementary Materials

The following are available online at https://www.mdpi.com/article/10.3390/brainsci11091218/s1, Figure S1: ROC curves of predictive models for ADHD diagnosis; Table S1: Evaluation of a binary system for ADHD prediction.
