# Supplementary material for: Impulsive and Omission Errors: Potential Temporal Processing Endophenotypes in ADHD
Source: Brain Sci. 2021 Sep 15;11(9):1218. doi: 10.3390/brainsci11091218 (PMC8467181; doi:10.3390/brainsci11091218)
Supplement: Supplementary file 1 [file brainsci-11-01218-s001.zip › brainsci-1335770-supplementary.pdf]

brain sciences

Supplementary Material

Impulsive and Omission Errors: Potential Temporal Processing Endophenotypes in ADHD

Johan E. Acosta-López[1,*,#], Isabel Suárez[2], David A. Pineda[3], Martha L. Cervantes-Henríquez[1,2], Martha L. Martínez-Banfi[1], Semiramis G. Lozano-Gutiérrez[1],Mostapha Ahmad[1], Wilmar Pineda-Alhucema[1], Luz M. Noguera-Machacón[1], Moisés De La Hoz[1], Elsy Mejía-Segura[1], Giomar Jimenez-Figueroa[1], Manuel Sánchez-Rojas[1], Claudio A. Mastronardi[4], Mauricio Arcos-Burgos[5,*], Jorge I. Vélez[2,*,#], Pedro J. Puentes-Rozo[1,6]

[1] Universidad Simón Bolívar, Facultad de Ciencias Jurídicas y Sociales, Barranquilla, Colombia; jacosta@unisimonbolivar.edu.co (J.E.A.L.), cervantesmh@unisimonbolivar.edu.co (M.L.C.H.), mmartinez108@unisimonbolivar.edu.co (M.L.M.B.), lozanosemiramis@gmail.com (S.G.L.G.), mostapha.ahmad@unisimonbolivar.edu.co (M.A.), wpineda1@unisimonbolivar.edu.co (W.P.A.), lnoguera1@unisimonbolivar.edu.co (L.M.N.M), mdelahoz48@unisimonbolivar.edu.co (M.D.L.H.), emejia18@unisimonbolivar.edu.co (E.M.S.), gdjimenez@unisimonbolivar.edu.co (G.J.F.), sanchezr@unisimonbolivar.edu.co (M.S.R.), ppuentes1@unisimonbolivar.edu.co (P.J.P.R.).

[2] Universidad del Norte, Barranquilla, Colombia; delchiaroi@uninorte.edu.co (I.S.), jvelezv@uninorte.edu.co (J.I.V).

[3] Neuropsychology and Conduct Research Group, University of San Buenaventura, Medellín, Colombia; david.pineda1@udea.edu.co (D.A.P.).

[4] INPAC Research Group, Fundación Universitaria Sanitas, Bogotá, Colombia; mastronardic@hotmail.com (C.A.M).

[5] Grupo de Investigación en Psiquiatría (GIPSI), Departamento de Psiquiatría, Instituto de Investigaciones Médicas, Facultad de Medicina, Universidad de Antioquia, Medellín, Colombia; mailto:mauricio.arcos@udea.edu.co (M.A.B.).

[6] *Grupo de Neurociencias del Caribe, Universidad del Atlántico, Barranquilla, Colombia.*

* *These authors contributed equally to this work.*

\# Correspondence: e-mail@e-mail.com.

Citation: Acosta-López, J.E.; Suárez, I.; Pineda, D.A.; Cervantes-Henriquez, M.L.; Martinez-Banfi, M.L.; Lozano-Gutierrez, S.G.; Ahmad, M.; Pineda-Alhucema, W.; Noguera-Machacón, L.M.; De La Hoz, M.; Mejía-Segura, E.; Jimenez-Figueroa, G.; Sánchez-Rojas, M.; Mastronardi, C.A.; Arcos-Burgos, M.; Vélez, J.I.; Puentes-Rozo, P.J. Impulsive and Omission Errors: Potential Temporal Processing Endophenotypes in ADHD . *Brain Sci.* **2021**, *11*, 1218. https://doi.org/10.3390/brainsci11091218

Academic Editor: Paul E. Engelhardt

Received: 27 July 2021
Accepted: 2 September 2021
Published: 15 September 2021

Publisher's Note: MDPI stays neutral with regard to jurisdictional claims in published maps and institutional affiliations.

Copyright: © 2021 by the authors. Submitted for possible open access publication under the terms and conditions of the Creative Commons Attribution (CC BY) license (https://creativecommons.org/licenses/by/4.0/).

Supplementary Table 1. Evaluation of a binary system for ADHD prediction. **(a)** Possible results when the real and predicted ADHD status are compared. Here, *a* is the number of individuals with ADHD that are correctly classified, *b* is the number of ADHD affected individuals classified unaffected (controls), *c* corresponds to the number of ADHD unaffected individuals (controls) classified as ADHD affected, and *d* to the number of ADHD unaffected individuals correctly classified. **(b)** Expressions for calculating the performance measures used to quantify the performance of the predictive model for ADHD.

(a)

Phenotype	Prediction	
	ADHD affected	Control
ADHD affected	*a*	*b*
Control	*c*	*d*

(b)

Measure	Expression
Sensitivity	*a* / (*a*+*c*)
Specificity	*d* / (*b*+*d*)
Precision	*a* / (*a*+*b*)
Classification rate (CR)	(*a*+*d*) / (*a*+*b*+*c*+*d*)
Lift	*a* (*a*+*b*+*c*+*d*) / {(*a*+*b*)(*a*+*c*)}

ADHD: Attention Deficit Hyperactivity Disorder.

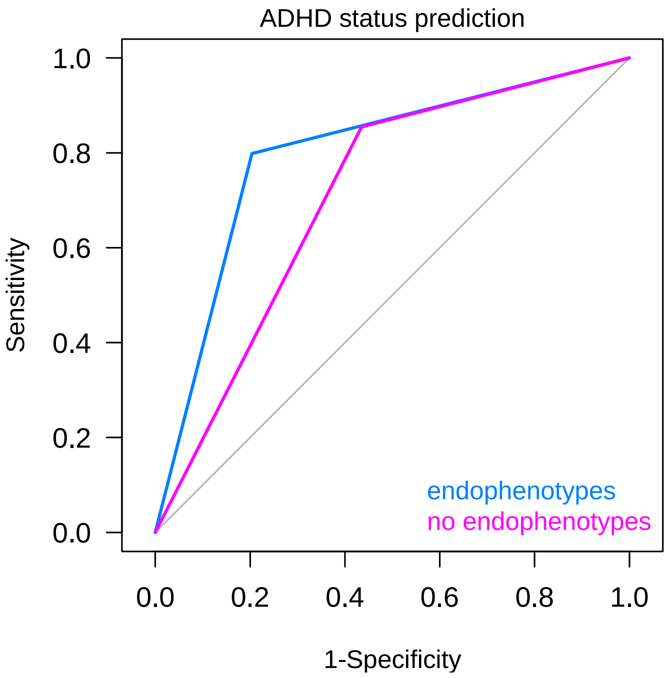

Supplementary Figure 1. ROC curves of predictive models for ADHD diagnosis including only demographic information (model 1, in pink) and endophenotypes (model 2, in blue). See Table 2 for more information on the neuropsychological tasks that met the endophenotypes criteria. Our results indicate that model 2 outperforms model 1 as measured by the specificity (0.796 vs. 0.565, $P<0.01$), classification rate (0.795 vs. 0.712, $P<0.05$), AUC (0.797 vs. 0.709, $P<0.05$) and lift (1.531 vs. 1.296, $P<0.05$) measures, and have a similar sensitivity (0.798 vs. 0.854, $P>0.05$). Overall, including the endophenotypes in the predictive model improves ADHD diagnosis classification compared to using demographic data alone.
